# Versatile Detection of Cellular Protein via Fluorescence Anisotropy

**DOI:** 10.1002/advs.76141

**Published:** 2026-07-13

**Authors:** Qing Tang, Yuan‐Ping Wei, Ricky Ruiqi Ma, Na Wei, Tammy Zihan Zhou, Hilary Kung‐Yu Ho, Zafar Iqbal Bhat, Iqra Ishrat, Xiaoyu Li, Judy Wai Ping Yam, Yung Hou Wong, Qiankun Wang, Justin L. Tan

**Affiliations:** ^1^ Institute of Cancer Research, High Throughput Screening Center Shenzhen Bay Laboratory Shenzhen China; ^2^ Division of Life Sciences and the Biotechnology Research Institute Hong Kong University of Science and Technology Hong Kong SAR China; ^3^ Department of Chemistry and State Key Laboratory of Synthetic Chemistry The University of Hong Kong Hong Kong SAR China; ^4^ Department of Pathology, School of Clinical Medicine The University of Hong Kong Hong Kong SAR China; ^5^ School of Basic Medical Sciences Capital Medical University Beijing China; ^6^ School of Chemical Biology and Biotechnology, Shenzhen Graduate School Peking University Shenzhen China; ^7^ School of Life Sciences University of Science and Technology of China Hefei China; ^8^ Laboratory for Synthetic Chemistry and Chemical Biology Limited, Health@InnoHK Innovation and Technology Commission Hong Kong SAR China; ^9^ State Key Laboratory of Nervous System Disorders and the Daniel and Mayce Yu Molecular Neuroscience Center Hong Kong University of Science and Technology Hong Kong SAR China; ^10^ InnoHK Hong Kong Center for Neurodegenerative Diseases Hong Kong SAR China; ^11^ Institute of Infectious Diseases Shenzhen Bay Laboratory Shenzhen China

**Keywords:** absolute protein quantification, biophysics, drug discovery, fluorescence anisotropy, high‐throughput screening, small molecule, target protein

## Abstract

Protein detection is a cornerstone of life science research. Here, we present Cell Lysate Fluorescence Anisotropy (CFAST), a versatile and accessible alternative cellular protein detection method. CFAST measures cellular protein levels through changes in fluorescence anisotropy of a long lifetime triangulenium dye‐labeled nanobody probe. The minimal steps of centrifugation and nuclease incubation remove unwanted cellular components. We demonstrate that CFAST robustly detects cellular PARP1, MK2, and GFP protein levels. Coupling small molecule incubation and thermal shift with CFAST (thermal shift‐CFAST) enables detection of cellular protein‐small molecule interactions through quantification of thermally stabilized target protein. Thermal shift‐CFAST successfully detects cellular PARP1 and MK2 inhibitor binding in a dose‐dependent manner. High‐throughput thermal shift‐CFAST screening identified a small molecule binder of undruggable transcription factor SOX2. This screening method also enabled development of a bifunctional PROTAC that simultaneously degrades membrane‐bound oncogene EGFR and inhibits immune modulating target HRH1. CFAST is rapid, taking a minimum of 2–3 hours to execute. It is also straightforward, utilizing common lab equipment and reasonably priced reagents. Our work suggests that CFAST is a viable alternative method to enhance protein detection as well as drug and probe discovery that is scalable, fast, and economical.

## Graphical abstract

Cell Lysate Fluorescence Anisotropy (CFAST) utilizes long lifetime dye‐labeled nanobodies for rapid protein quantification in minimally purified, complex cell lysate. When coupled with cellular thermal shift, CFAST allows high‐throughput detection of endogenous protein‐small molecule engagement, facilitating the discovery of novel binders for Proteolysis Targeting Chimera (PROTAC) discovery. CFAST is a versatile and accessible method for protein quantification as well as probe and drug discovery. Created with BioRender.

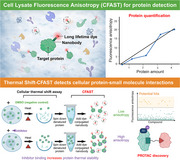

## 1 Introduction

Proteins play key roles in numerous biological processes. Accurate protein detection is therefore a crucial process in understanding biology. Western blotting is the conventional protein quantification technique employed by nearly all research labs engaged in biological research. This method requires multiple sequential steps, including sample preparation, electrophoretic separation, membrane transfer, blocking, antibody incubation, washing, detection, and densitometric analysis [1, 2]. While relatively straightforward to execute, the multiple steps involved could introduce inadvertent experimental error. These steps also require significant time commitment. Another disadvantage is the low‐throughput nature of Western blotting. Conversely, a commonly employed high throughput alternative for protein detection is mass spectrometry (MS). Proteins purified from cell lysates are digested into peptides, separated by liquid chromatography (LC), then analyzed by a mass spectrometer to quantify specific peptides corresponding to the proteome [3]. LC‐MS is particularly useful in detecting a large range of proteins from individual samples, but the relatively high cost for assessing large numbers of biological samples and high technical barrier restricts everyday use by most labs [4]. There is room for developing alternatives to conventional protein detection methods.

Proteolysis Targeting Chimeras (PROTACs) are a revolutionary new drug modality that channel target proteins for degradation [5]. PROTACs are bifunctional small molecules comprising two moieties, an E3 ligase binder covalently linked to a binder of a protein target of interest, also known as the warhead. These molecules bring the target protein in close proximity to the E3 ligase, promoting targeted ubiquitination of the protein. This marks the protein for proteasomal degradation [6]. Since this degradation is independent of protein activity, warheads can be designed to bind protein regions outside of conventional active sites or binding pockets. This increases the repertoire of proteins that can be drugged by degradation rather than traditional inhibition [7]. However, current efforts on PROTAC development have focused on already drugged proteins [8]. This suggests that discovering and developing warheads against undruggable proteins remains a challenge. A reason for challenging warhead discovery against undruggable proteins is the lack of suitable assays. Various biochemical assays have been developed to identify protein‐small molecule binding [9], but most are performed in vitro. These assays require costly instrumentation as well as purified proteins, and do not assess cell permeability or biological activity. In contrast, cell‐based assays validate these aspects but often fail to confirm cellular target engagement [10].

Methods that detect cellular protein‐small molecule interactions have been recently developed to address this bottleneck in drug discovery. The Cellular Thermal Shift Assay (CETSA) measures ligand‐induced thermal stabilization of proteins in cells, typically by Western blotting [11]. To improve scalability, adaptations have been developed including AlphaLISA‐based CETSA screening [12] and split NanoLuc‐based CETSA assays, which enable luminescence readout in multi‐well formats [13]. Additionally, mass spectrometry‐based thermal proteome profiling (TPP) enables proteome‐wide analysis of thermal stability shifts with small molecule screening [14, 15, 16]. However, each approach has limitations: CETSA screening requires specific antibody pairs against protein dimers, limiting its utility against many monomeric proteins. Luciferase‐based methods rely on protein tagging and therefore require genetic modification of cells. Proteomics approaches require specialized instrumentation and complex analysis, resulting in high costs and technical barriers that limit widespread utility. These limitations highlight the need for alternative assays that are low‐cost, scalable, and straightforward for the average research group.

To provide an alternative versatile, scalable, efficient, and accessible method for protein detection, we developed Cell Lysate Fluorescence Anisotropy (CFAST). CFAST innovates on fluorescence anisotropy (FA) and altered protein thermal stability by small molecule binding. FA measures plane‐polarized fluorescence emission differences in perpendicular planes when a fluorescent molecule tumbles at different rates [17]. This method typically assesses binding between a protein‐of‐interest and a dye‐labeled small molecule probe. Higher FA, measured in milli‐polarization units (mP), is proportional to an increase in molecular mass of the probe complex [18]. However, FA is typically performed in vitro due to a high background caused by viscosity of more complex solutions [19]. In addition, FA is limited to targets with known small molecule binders to act as probes. Probes are typically of smaller masses to increase the sensitivity of detection [20]. To overcome the aforementioned limitations, we employed long lifetime triangulenium dye‐labeled nanobody probes [21, 22, 23], as well as nuclease incubation and centrifugation steps, to enable FA to quantify native, soluble cellular protein in lysates. CFAST robustly detects endogenous PARP1, MK2, and GFP protein levels after a short 3‐hour protocol. Additionally, CFAST enables absolute quantification of cellular PARP1 using known standards. Our work suggests that CFAST can be employed as a robust, practical protein detection method.

CFAST detects cellular protein‐small molecule interactions by coupling with CETSA [24], named thermal shift‐CFAST. When cell permeable small molecules bind intracellular protein targets, they thermally stabilize them. In CETSA, this stabilization is detected via heating to denature unbound proteins, centrifugation to remove denatured proteins, and finally quantification of the remaining soluble protein which is stabilized by binding [24]. In thermal shift‐CFAST, CFAST replaces the traditional Western blot protein quantification employed by CETSA. We demonstrate that thermal shift‐CFAST identifies PARP1 inhibitor olaparib and MK2 inhibitor PF‐3644022 interactions with their endogenous target proteins in a dose‐response manner. Next, we optimized thermal shift‐CFAST for high‐throughput screening in 384‐well plates in less than 10 hours, screening a library of ≈900 approved drugs, active pharmaceutical ingredients, and synthetic compounds on HEK293T cells expressing tagged SOX2 or EGFR. SOX2 is an undruggable transcription factor while EGFR is a membrane‐bound oncogene. We identified wedelolactone as a novel SOX2 binder. In addition, we also identified HRH1 inhibitor desloratadine as an EGFR binder. From this binder, we developed a bifunctional PROTAC that simultaneously degrades EGFR and inhibits HRH1, which has recently been shown to modulate an immune checkpoint [25]. Our results indicate that CFAST is a scalable, practical, and broadly applicable technique that could assist PROTAC development against novel targets.

**FIGURE 1:**
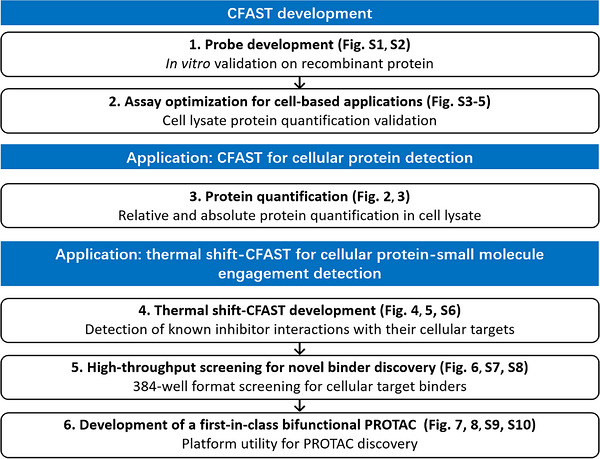
CFAST development and application roadmap.

Overall, our work details the development and utility of CFAST for versatile protein quantification applications (Figure 1). First, we developed and validated the utility of long lifetime dye‐ and nanobody‐based FA probes for quantifying proteins of interest in complex cell lysate. Second, we integrated CETSA with CFAST (thermal shift‐CFAST) for large‐scale chemical screening to identify cellular protein‐small molecule interactions. Last, we demonstrate the practical utility of this platform by developing an identified ligand into a bifunctional PROTAC. We propose CFAST as a versatile, practical, and accessible tool for rapid protein quantification as well as probe and drug discovery applications.

## 2 Results

### 2.1 Long Lifetime Fluorescent Dye Labeled Nanobody Probes can Detect Cellular Protein Levels

To establish a robust FA‐based readout for detecting proteins in cell lysate, we first evaluated key design parameters that influence FA dynamic range. FA is dependent on both the rotational correlation time of the probe and the fluorescence lifetime of the fluorophore [26]. We therefore examined the impact of probe size and fluorophore properties on assay performance. Antibodies and more recently, nanobodies (VHH), have been shown to possess high specificity binding to target proteins [27]. By modeling from Perrin's theory of FA [28], a nanobody‐based probe coupled with a long lifetime (20 ns) fluorescent dye is expected to yield an improved dynamic range in anisotropic change (ΔmP) for protein detection up to a molecular weight of 200–300 kiloDaltons (kDa) compared to a conventional antibody (Figure S1A). We selected commercially available diazaoxatriangulenium (DAOTA), a long lifetime (≈20 ns) fluorescent dye with excitation and emission maxima at approximately 550 nm and 590 nm for testing (Figure S1B). Consistent with the Perrin theory prediction, DAOTA‐labeled nanobody probes exhibited significantly greater FA changes upon binding to recombinant MK2 compared to an antibody‐based probe, that showed no discernable difference (Figure S1C). Our data suggests that a DAOTA‐labeled nanobody probe is suitable for FA‐based protein detection.

To validate this detection method against other proteins, we synthesized DAOTA‐labeled nanobody probes targeting PARP1 and MK2, with GFP as a control probe for non‐specific binding. We tested all probes with their respective recombinant protein targets. Significant, dose‐dependent changes in FA were observed when PARP1 probe was incubated with recombinant PARP1 protein, but not with GFP probe (Figure S2A). We also observed a similarly significant response for MK2 probe incubated with recombinant MK2 protein, but not when PARP1 and GFP probes were used (Figure S2B). These results suggest an acceptable degree of in vitro specificity of our probes.

Next, we devised a protocol that enabled our probes to detect cellular protein with the minimum number of steps. Freeze‐thaw cycles were used to lyse cells, followed by centrifugation to remove insoluble material. Probe was then incubated with the resultant supernatant, after which FA was measured. We tested this protocol to detect cellular MK2 in wildtype (WT) HEK293T cells and HEK293T cells overexpressing MK2. To minimize effects from non‐specific probe binding, we added a control GFP probe to every sample for normalization. Initial results showed high background in our FA readings compared to in vitro results (Figures S2A,B, S3). We suspected that this was due to the presence of soluble nucleic acids which contributed to an unacceptably high viscosity of the solution. By adding an incubation step with a nuclease that digests both DNA and RNA, we significantly reduced the background FA, allowing for robust quantification of MK2 (Figure S3). Our results suggest that nuclease incubation is recommended to reduce solution viscosity and increase the dynamic range of the FA readout. Based on our data thus far, we affirmed a workflow for cell lysate‐based protein quantification via FA (CFAST) (Figure S4).

### 2.2 CFAST can Quantify Absolute Cellular Protein

**FIGURE 2:**
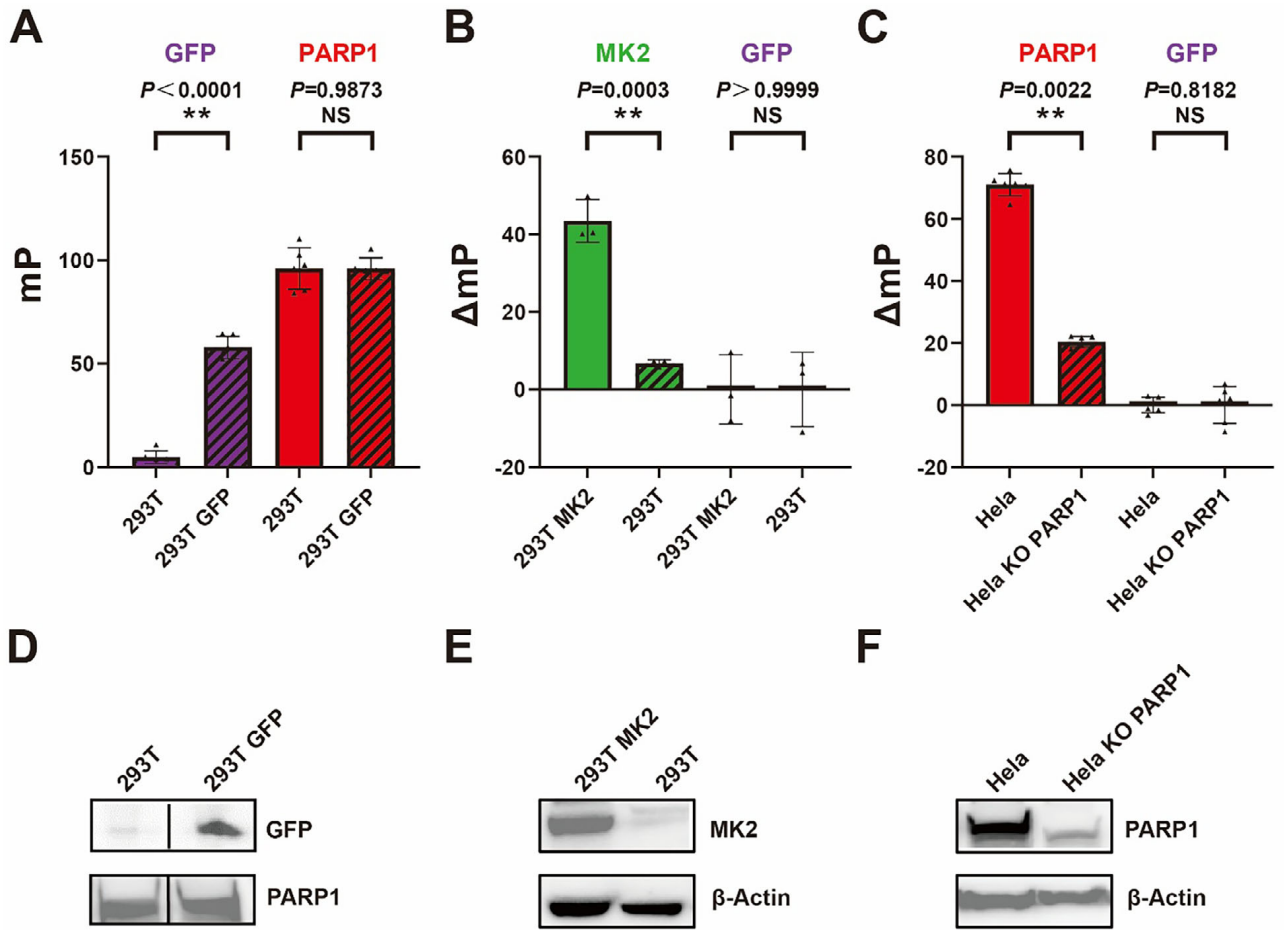
CFAST can quantify cellular protein levels. (A) FA measurements of GFP and PARP1 probes in HEK293T and HEK293T expressing GFP cell lysates. ΔmP values were obtained by normalizing to the HEK293T‐GFP probe sample. Data are presented as mean ± SD, n = 5. (B) FA measurements of MK2 and GFP probes in HEK293T and HEK293T expressing MK2 cell lysates. ΔmP values were obtained by normalizing to mP values of the GFP probe samples. Data are presented as mean ± SD, n = 3. (C) FA measurements of PARP1 and GFP probes in Hela and Hela PARP1 knockout cell lysates. Data are presented as mean ± SD, n = 5. ΔmP values were obtained by normalizing to mP values of the GFP probe samples. Western blots for (D) GFP and PARP1 in HEK293T and HEK293T expressing GFP cells, (E) MK2 and ACTB in HEK293T expressing MK2 and HEK293T cells, and (F) PARP1 and ACTB in Hela and Hela PARP1 knockout cells. Statistical significance was determined using a two‐tailed unpaired Student's *t*‐test.

We applied our optimized CFAST protocol as an alternative to immunoblotting to detect cellular protein levels of GFP, PARP1, and MK2 protein. In HEK293T and HEK293T expressing GFP cells, CFAST could robustly detect high levels of GFP in the GFP‐expressing line, with no changes observed in PARP1 (Figure 2A). We also observed the expected trend of high FA signal for MK2 in MK2 overexpressing cells and low PARP1 signal in knockout (KO) cells compared to controls, with minimal detection in the GFP control probe samples (Figure 2B,C). We validated that protein levels in the various cell lines by Western blot, which tallied with their respective CFAST readouts (Figure 2D–F). These results suggest that CFAST can detect changes in cellular protein levels.

Since triangulenium dyes are not as commonly used as conventional short lifetime fluorophores such as FITC, we examined the possibility of utilizing FITC‐labeled nanobody probes in CFAST. Perrin's theory predicts that a longer lifetime fluorescent dye like DAOTA (≈20 ns) would demonstrate a better dynamic range of detection, across a board range of molecular weights, compared to the commonly used short lifetime (4 ns) FITC (Figure S5A). To assess this, we tested a FITC‐labeled nanobody probe against PARP1, using a FITC‐GFP probe as background control (Figure S5B). The FITC‐labeled nanobody probe for PARP1 showed similar signal to background FA as defined by the FITC‐GFP signals, regardless of PARP1 KO in cells (Figure S5B). This contrasts with DAOTA probe data, where a significant differential FA signal is observed relative to PARP1 levels in the Hela WT and PARP1 KO cells (Figure 2C). Our data suggests that only probes with sufficiently long lifetimes like DAOTA can be utilized for CFAST.

**FIGURE 3:**
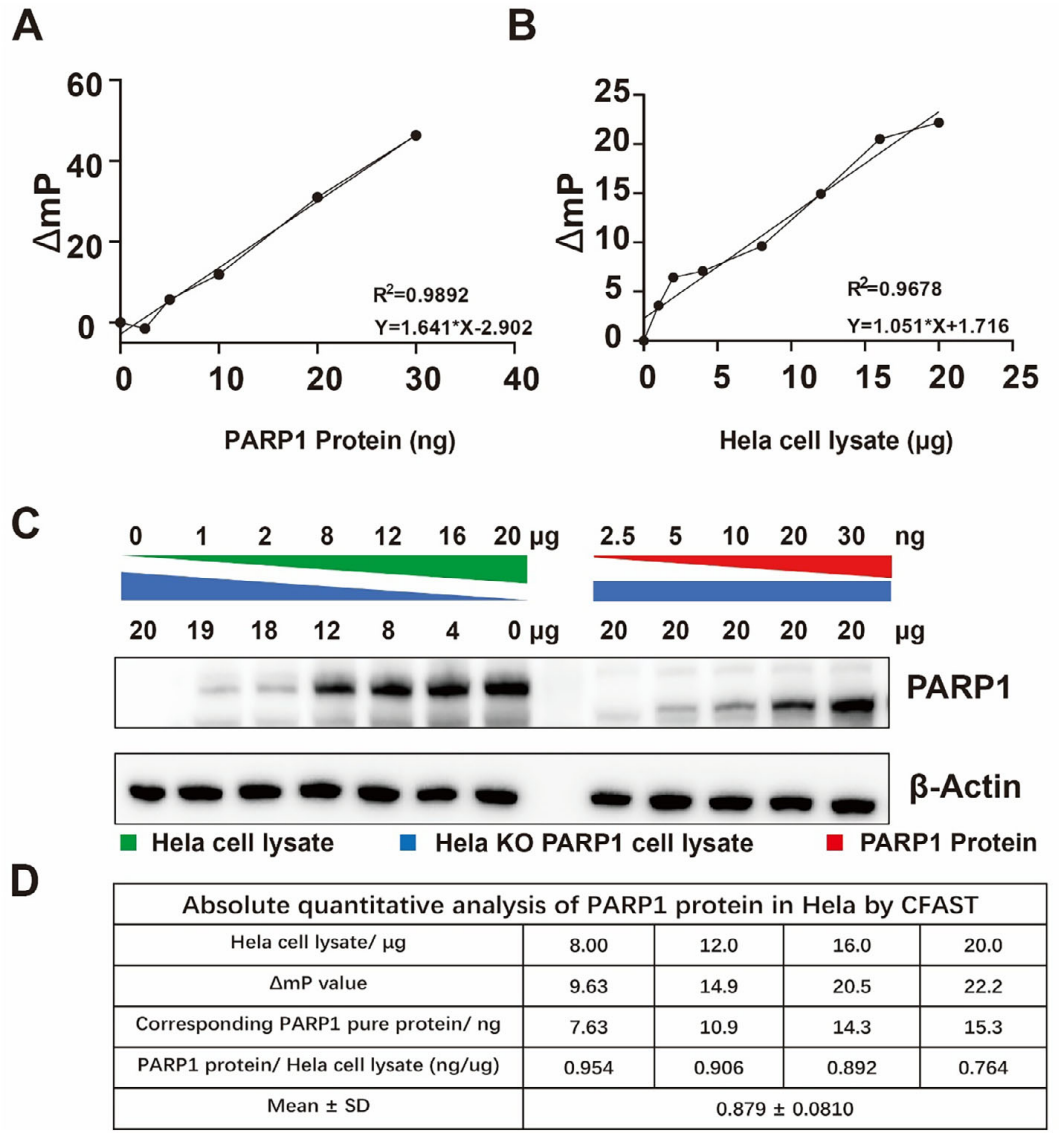
CFAST can quantify absolute amounts of cellular PARP1. (A) FA measurements of PARP1 probe in varying amounts of recombinant PARP1 protein added to the same amount of Hela PARP1 knockout cell lysate. (B) FA measurements of PARP1 probe in increasing amounts of Hela cell lysate balanced with decreasing amounts of Hela PARP1 knockout lysate. (C) Western blot for PARP1 and ACTB in increasing amounts of Hela cell lysate balanced with decreasing amounts of Hela PARP1 knockout lysate, and in increasing amounts of recombinant PARP1 protein balanced with the same amount of Hela PARP1 knockout cell lysate. (D) Tabulation of values used to determine the absolute amount of PARP1 in Hela cell lysate. ΔmP values were obtained by normalizing to FA measurements of GFP probe added to aliquots of the experimental samples. The reported mean ± SD (0.879 ± 0.081 ng PARP1 per µg HeLa cell lysate) was calculated from 4 independent calibration‐based estimates derived from the indicated lysate input amounts.

As our CFAST measurements vary proportionally with the amount of protein target, we examined if this method could be used for absolute protein quantification. To quantify the amount of PARP1 protein in Hela cells, we first generated a calibration curve using varying amounts of recombinant PARP1 protein added to equal amounts of Hela KO PARP1 cell lysate (Figure 3A). The 0–30 ng recombinant protein range was selected to approximate the pre‐saturation response window (≈0–18 nM) of the in vitro PARP1 response curve (Figure S2A). The presence of Hela lysate devoid of PARP1 in the standard curve measurements accounts for the background viscosity due to the cellular composition of Hela cells. This allows for a more accurate comparison to CFAST measurements of endogenous PARP1 in WT Hela cells (Figure 3B). For these endogenous measurements however, adding more Hela cells would alter the background cellular composition of each measurement. Hence, similar to the standard curve, we added decreasing amounts of Hela KO cell lysate to increasing amounts of WT Hela lysates. This ensured a balanced cellular composition, with the only variable being the amount of endogenous PARP1 protein (Figure 3C). Based on our readings, we calculated the absolute amount of PARP1 protein as ≈0.879 ± 0.0810 ng/µg of Hela cell lysate (Figure 3D). This result closely aligns with previous findings from a study that utilized MS for quantification [29], validating the acceptable accuracy of our results. Our data suggest that CFAST can robustly determine the relative and absolute abundance of cellular proteins‐of‐interest.

### 2.3 CFAST Combined with Thermal Stability Enables Cellular Protein‐Small Molecule Interaction Detection

**FIGURE 4:**
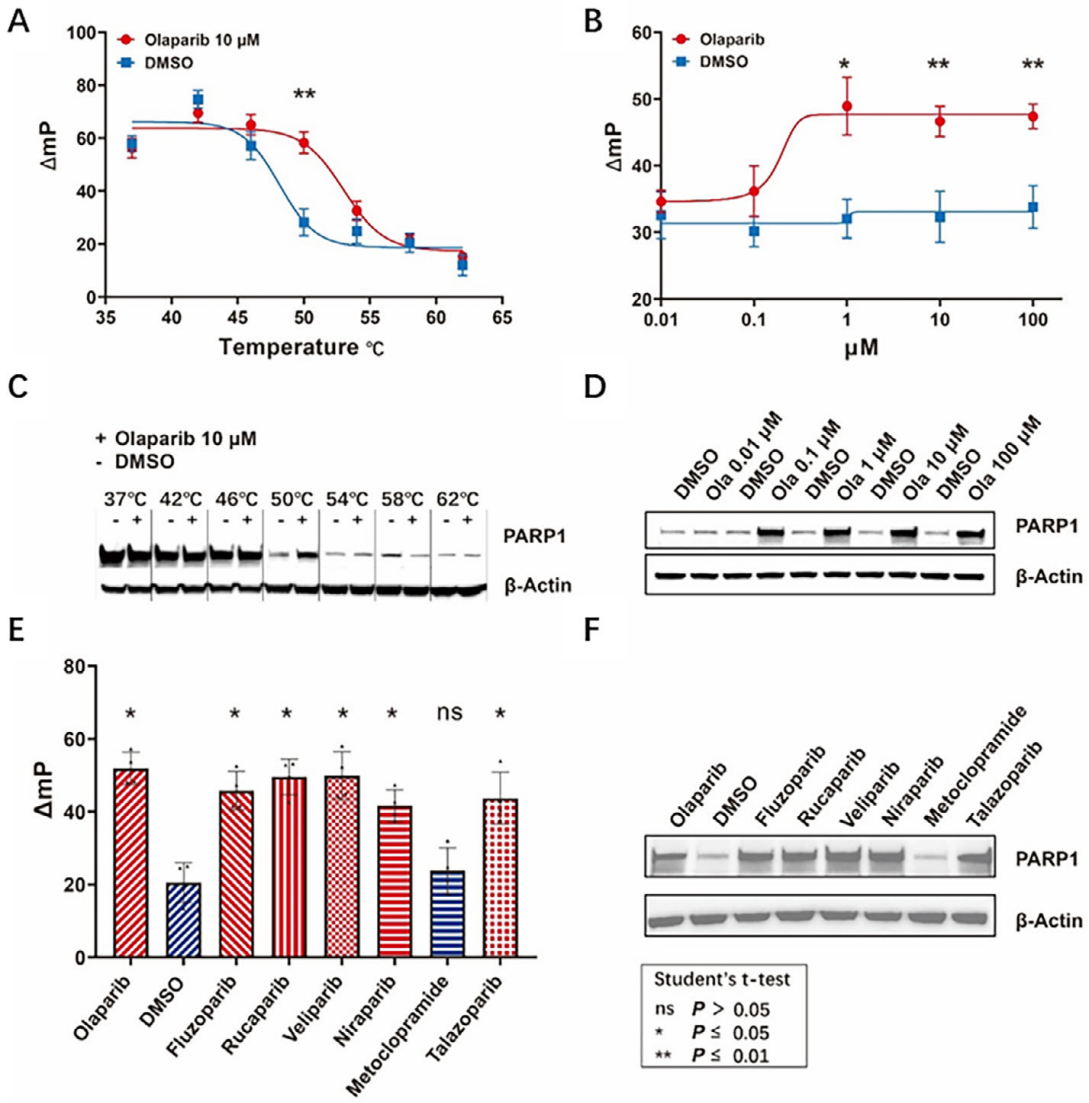
CFAST detects binding of known PARP1 inhibitors to cellular PARP1. (A) CFAST measurements of PARP1 probe in DMSO‐ and olaparib‐treated HEK293T cell lysate, heated at various temperatures. Data are presented as mean ± SD, n = 5. (B) Dose‐response CFAST measurements of PARP1 probe in DMSO‐ and olaparib‐treated HEK293T cell lysate, heated at 50°C. Data are presented as mean ± SD, n = 5. (C) CETSA Western blot of PARP1 and ACTB in DMSO‐ and olaparib‐treated HEK293T cells, heated at various temperatures. (D) Dose‐response CETSA Western blot of PARP1 and ACTB in DMSO‐ and olaparib‐treated HEK293T cells, heated at 50°C. (E) CFAST measurements of PARP1 in HEK293T cells incubated with DMSO and metoclopramide negative controls, and known PARP1 inhibitors, heated at 50°C. P‐values are calculated relative to the DMSO group. Data are presented as mean ± SD, n = 4. (F) CETSA Western blot for PARP1 and ACTB in HEK293T cells treated with the same drugs in **E**, heated at 50°C. ΔmP values were obtained by normalizing to CFAST measurements of GFP probe added to aliquots of experimental samples. Statistical significance was determined using a two‐tailed unpaired Student's *t*‐test.

**FIGURE 5:**
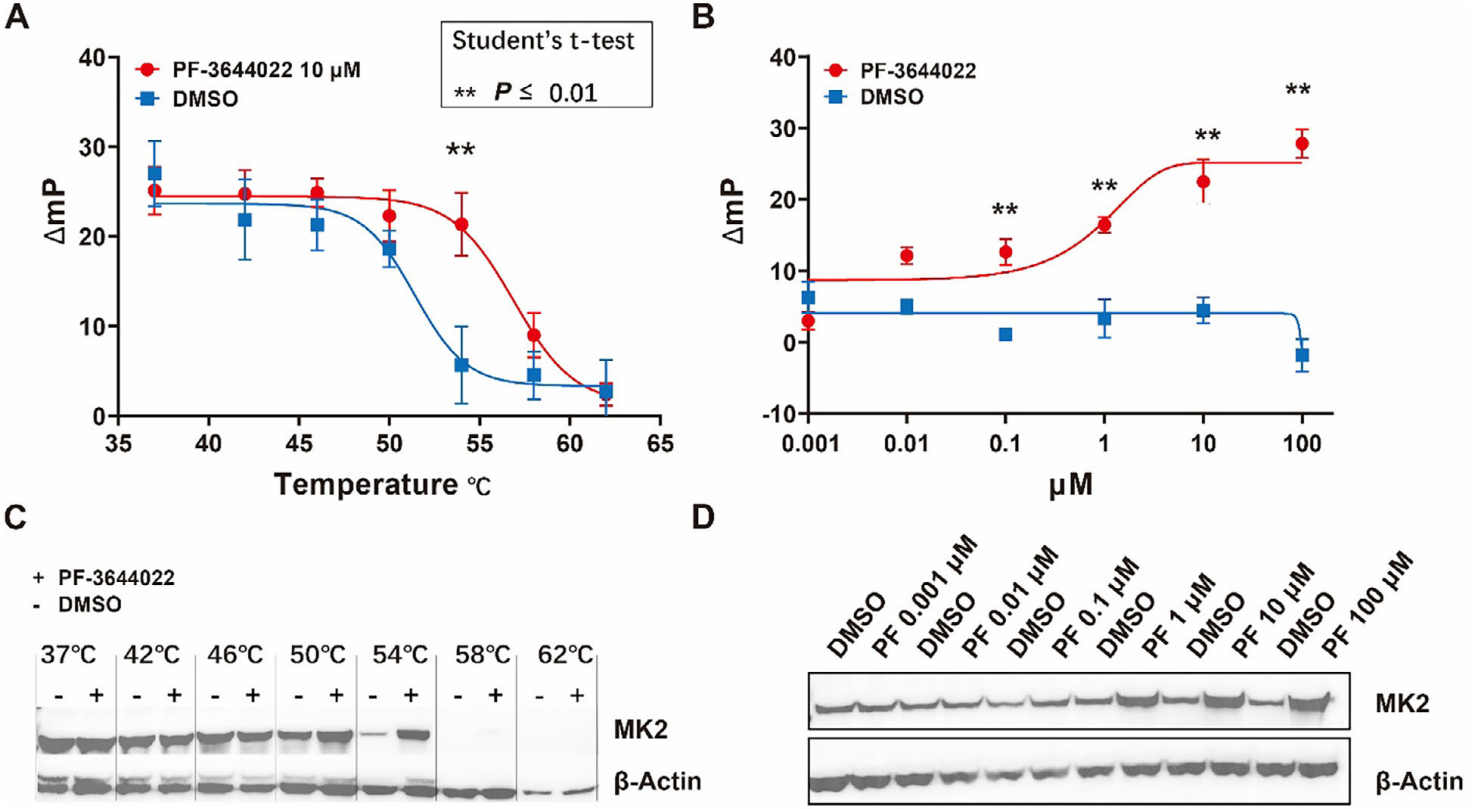
CFAST detects binding of an MK2 inhibitor to cellular MK2. (A) CFAST measurements of MK2 probe in DMSO‐ and PF‐3644022‐treated HEK293T expressing MK2 cell lysate, heated at various temperatures. (B) Dose‐response CFAST measurements of MK2 probe in DMSO‐ and PF‐3644022‐treated HEK293T expressing MK2 cell lysate, heated at 54°C. (C) CETSA Western blot of MK2 and ACTB in DMSO‐ and PF‐3644022‐treated HEK293T expressing MK2 cells, heated at various temperatures. (D) Dose‐response CETSA Western blot of MK2 and ACTB in DMSO‐ and PF‐3644022‐treated HEK293T expressing MK2 cells, heated at 54°C. ΔmP values were obtained by normalizing to CFAST measurements of GFP probe added to aliquots of experimental samples. Data are presented as mean ± SD, n = 5. Statistical significance was determined using a two‐tailed unpaired Student's *t*‐test.

To enable CFAST detection of cellular protein‐small molecule interactions, we coupled CETSA, a well‐established method for assessing protein–ligand interactions in cells, to CFAST to yield a protocol for thermal shift‐CETSA (Figure S6). This allows for drug incubation in live cells to engage and stabilize target proteins, such that upon heating‐induced denaturation, a greater fraction of ligand‐bound proteins remains in the soluble native state. Optional nuclease treatment and centrifugation are applied to reduce solution viscosity and remove insoluble material. Instead of a Western blot readout as used in conventional CETSA, CFAST is applied to quantify the amount of stabilized protein, serving as a proxy for cellular target engagement. We applied this modified protocol to live HEK293T cells incubated with a fixed concentration of known PARP1 inhibitor olaparib and processed at different temperatures. CFAST detected a significant increase in the thermal stability of cellular PARP1 owing to olaparib binding (Figure 4A). In addition, CFAST could quantify the interaction between cellular PARP1 and olaparib in a dose‐dependent manner (Figure 4B). CETSA performed in a similar temperature gradient and dose‐response fashion for PARP1 and olaparib recapitulated our CFAST results (Figure 4C,D). Further, we tested 6 additional PARP1 inhibitors and demonstrated significant CFAST detection of their association with cellular PARP1, in concordance with CETSA (Figure 4E,F). DMSO and non‐PARP1 binder metoclopramide expectedly showed negative results. To assess the applicability of small molecule binding detection by CFAST to other proteins, we treated HEK293T cells overexpressing MK2 with known MK2 inhibitor PF‐3644022. Similar to PARP1, CFAST was able to detect increased thermal stability of cellular MK2 with PF‐3644022 treatment (Figure 5A) and dose‐dependent binding of PF‐3644022 to MK2 (Figure 5B). These MK2 results were comparable with CETSA (Figure 5C,D). Our results suggest that CFAST is a viable alternative for detecting cellular protein‐small molecule interactions.

### 2.4 CFAST Screening Identifies a Small Molecule Binder of Undruggable Transcription Factor SOX2

**FIGURE 6:**
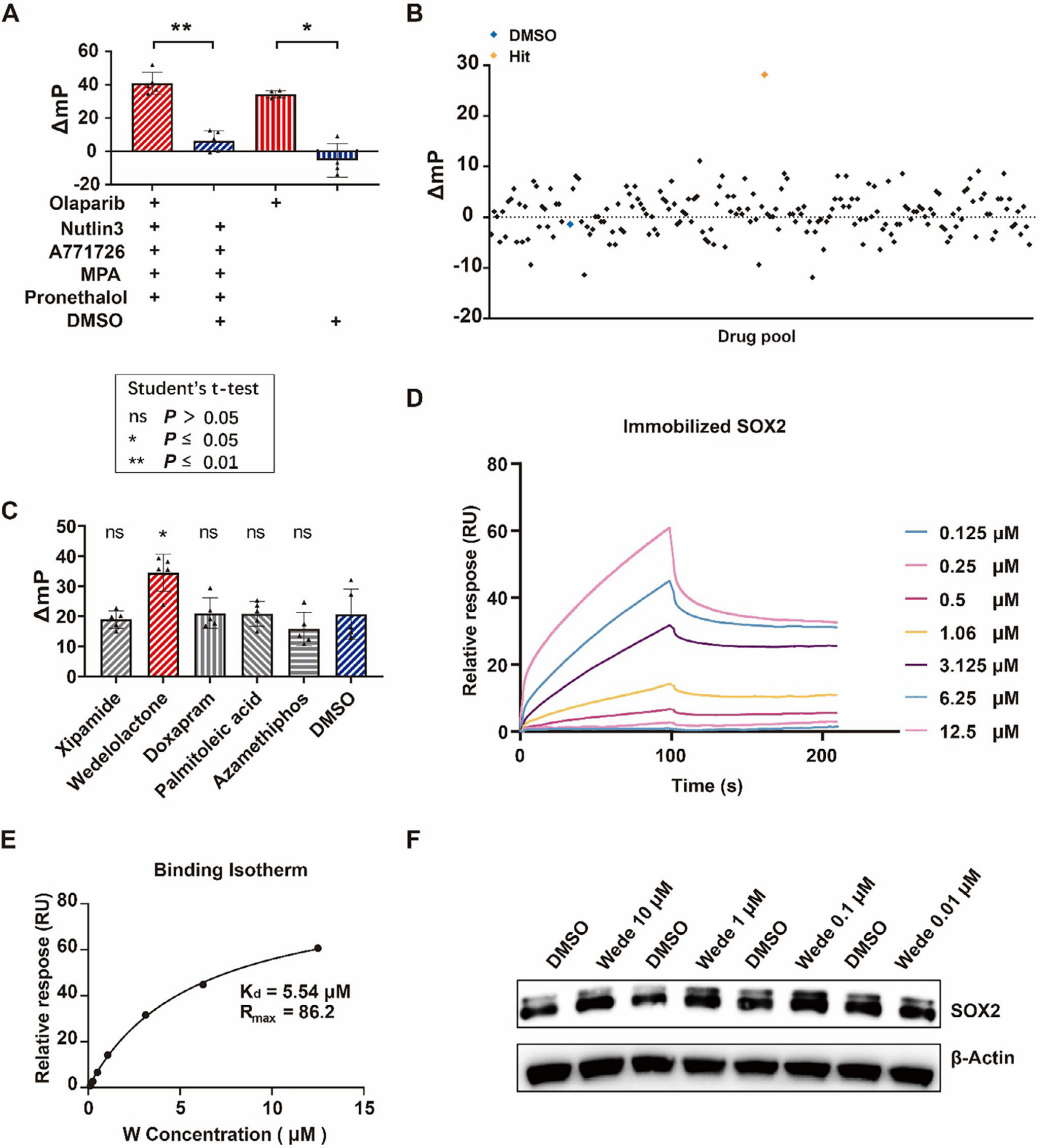
CFAST screening identifies a novel small molecule binder of SOX2. (A) CFAST measurements of PARP1 probe in HEK293T cells incubated with 10 µM of various small molecule combinations, heated at 58°C. Data are presented as mean ± SD. For panel A, the first group (Olaparib) n = 5, and the second group (Nutlin3/A771726/MPA/Pronethalol/DMSO) n = 4. (B) High throughput CFAST measurements of MYC‐tag probe in HEK293T cells expressing MYC‐tag SOX2 incubated with 900 small molecules in pools of 5, heated at 58°C. Values are the average of 2 replicates, n = 2. (C) CFAST for SOX2 in HEK293T *Tg:SOX2* cells treated with drugs from the pooled hit at 10 µM, heated at 58°C. P‐values are calculated relative to the DMSO group. Data are presented as mean ± SD, n = 5. (D, E) Dose‐response surface plasmon resonance measurements of wedelolactone and recombinant SOX2 protein interaction. ΔmP values were obtained by normalizing to CFAST mP measurements of GFP probe added to an aliquot of experimental samples. (F) Dose‐response CETSA Western blot of SOX2 and ACTB in DMSO‐ and wedelolactone‐treated H69 cells, heated at 58°C. (A, C) Statistical significance was determined using a two‐tailed unpaired Student's *t*‐test.

We next investigated if CFAST could be scaled for high‐throughput screening. First, we tested if CFAST could identify a positive hit from a pool of 5 small molecules. Expectedly, CFAST robustly detected PARP1‐olaparib binding in HEK293T cells treated with a pool of olaparib combined with four other drugs, to a similar degree as olaparib treatment alone (Figure 6A). Next, we adapted our protocol for 384‐well format, scaling down reagents and utilizing a lower centrifugation speed (Figure S7).

We then applied CFAST to screen for small molecule binders of presently undruggable oncogene SOX2 [30]. SOX2 is a Yamanaka transcription factor that plays a pivotal role in maintaining pluripotency and self‐renewal in stem cells [31]. SOX2 is also implicated in various cancers, where it contributes to tumorigenesis and cancer progression by regulating key signaling pathways [32]. We screened a library of 900 approved drugs and active pharmaceutical ingredients in pools of 5 drugs on HEK293T cells expressing MYC‐tagged SOX2. In the absence of commercially available nanobodies against SOX2, we adopted an epitope‐tagging strategy and utilized a 2 × MYC tag, a small tag (≈2.4 kDa) that minimally perturbs protein structure and function. A high‐affinity MYC‐tag nanobody (Kd ≈ 0.5 nM) labeled with DAOTA was used as the probe for CFAST detection. We obtained one hit pool, and further CFAST testing of the individual drugs in the pool identified wedelolactone as a SOX2 binder (Figure 6B,C). We validated that wedelolactone binds SOX2 with a K_d_ of 5.54 µM via surface plasmon resonance (Figure 6D,E). In addition, CETSA revealed that wedelolactone increased the thermal stability of SOX2 in the H69 cell line, which endogenously expresses SOX2 (Figure 6F). Wedelolactone, which is derived from the plant wedelia chinensis, has been recognized for its anti‐inflammatory and anti‐cancer properties [33]. However, there is currently no evidence of wedelolactone binding directly to SOX2. Our data suggests that CFAST can be used to robustly screen for small molecule binders of protein targets, particularly undruggable nuclear targets like SOX2, which can be useful in drug and probe discovery and development.

### 2.5 CFAST Screening Identifies a Small Molecule Binder of Membrane‐Bound Oncogene EGFR

**FIGURE 7:**
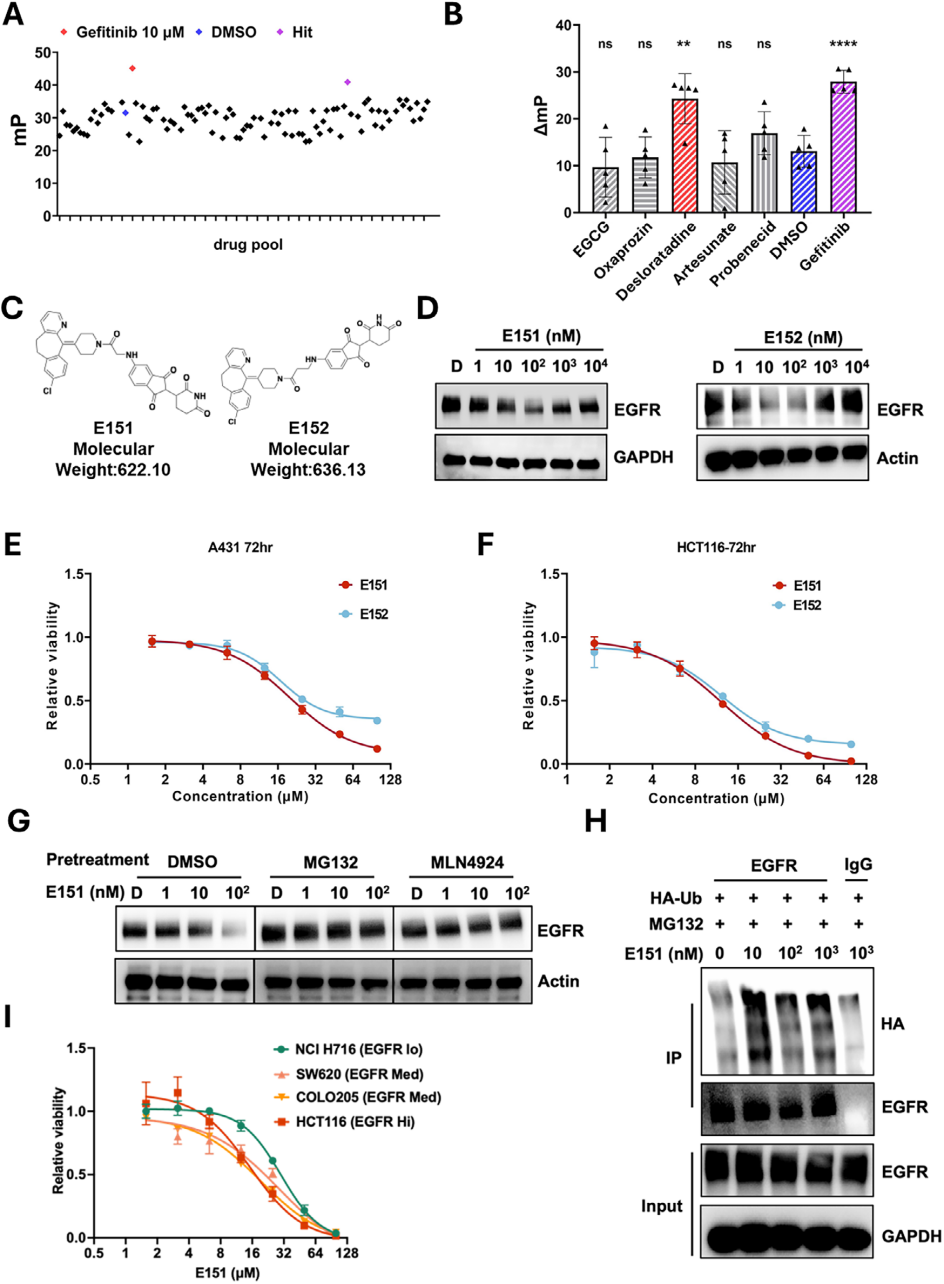
Desloratadine warhead PROTACs target EGFR. (A) High throughput CFAST measurements of MYC‐tag probe in HEK293T cells expressing MYC‐tag EGFR incubated with 400 small molecules in pools of 5, heated at 50°C. (B) CFAST for EGFR in HEK293T cells expressing MYC‐tag EGFR treated with drugs from the pooled hit at 10 µM, heated at 50°C. P‐values are calculated relative to the DMSO group. Data are presented as mean ± SD, n = 5. (‐)‐Epigallocatechin Gallate (EGCG), a compound found in the hit drug pool, is included as one of the tested drugs. Statistical significance was determined using a two‐tailed unpaired Student's *t*‐test. (C) Chemical structures of PROTACs E151 and E152 with desloratadine warheads. (D) Dose‐response western blot of EGFR, GAPDH, and Actin in DMSO‐, E151‐ and E152‐treated A431 cells. Cell viability of (E) A431 and (F) HCT116 cells treated with E151 or E152 for 72 hrs, measured by CellTiter‐Glo assay. Data are presented as mean ± SD, n = 3. (G) Dose‐response western blot of EGFR and Actin of DMSO‐ and E151‐treated A431 cells pretreated with DMSO, proteasome inhibitor MG132, or neddylation inhibitor MLN4924. (H) Immunoprecipitation of EGFR and HA‐tagged ubiquitin in A431 cells treated with MG132 and E151. GAPDH serves as loading control. (I) Cell viability of E151‐treated colorectal cancer cell lines with variable EGFR expression, measured by CellTiter‐Glo assay. Data are presented as mean ± SD, n = 3.

Since we showed that CFAST can discover binders of an undruggable nuclear protein, we wondered if our method could be useful for membrane protein drug or probe discovery. EGFR is an important membrane‐bound protein that triggers oncogenic signaling in cancer. Wildtype and mutant EGFR have been successfully drugged in various cancers, but drug resistance continues to be an issue [34, 35]. Novel compounds could be useful in developing next‐generation EGFR drugs. We therefore screened our compound library on HEK293T cells expressing MYC‐tagged EGFR using EGFR inhibitor gefinitib as control. Like our SOX2 screen, a suitable EGFR nanobody was commercially unavailable, so we utilized a MYC‐tag nanobody probe and MYC‐tagged EGFR expressing cell line for the EGFR screen. Unexpectedly, we identified one hit pool (Figure 7A). Further CFAST testing of the individual drugs in the pool identified desloratadine as a previously uncharacterized EGFR binder (Figure 7B). We validated that desloratadine binds EGFR with ≈677 nM half maximal inhibitory concentration (IC_50_) via fluorescence polarization (Figure S8A). Furthermore, temperature gradients and dose‐response CETSA experiments with desloratadine treatment revealed that this drug stabilizes cellular EGFR (Figure S8B–D). These data suggest that CFAST successfully identified desloratadine as a novel EGFR binder. CFAST is therefore applicable to membrane protein drug and probe discovery.

### 2.6 Development of a Bifunctional EGFR/HRH1 Targeting Tool Compound to Simultaneously Probe Cancer Signaling and Immune Checkpoint Inhibition

While desloratadine, the active metabolite of loratadine, is widely used as an antihistamine targeting histamine receptor H1 (HRH1), a recent study concluded that antihistamines targeting HRH1 on tumor‐associated macrophages (TAMs) could alleviate immunotherapy resistance in cancer patients [25]. Hence, we wondered if we could repurpose desloratadine to develop a bifunctional PROTAC targeting EGFR and HRH1 to simultaneously probe oncogenic signaling and the tumor microenvironment. We synthesized two PROTAC analogs E151 and E152 with a desloratadine warhead and a pomalidomide E3 ligand moiety (Figure 7C), Both compounds demonstrated dose‐dependent depletion of endogenous EGFR in the A431 epidermoid carcinoma cell line, with the Hook effect observed at higher doses (Figure 7D). EGFR degradation was rapid, with significant reduction observed within 6 hours, and highly potent, with estimated half maximal degradation concentrations (DC_50_) of 94.4 nM for E151 and 8.63 nM for E152 (Figure 7D). The corresponding parent warhead desloratadine did not induce detectable EGFR degradation in either A431 or HCT116 cells under the same experimental conditions (Figure S9A,B), suggesting that EGFR degradation is dependent on the PROTAC design rather than the warhead alone. Our results suggest that desloratadine‐based PROTACs can potently degrade EGFR.

We next examined if our PROTACs had biological effects in cancer cell lines. Consistent with their EGFR‐degrading activity, both compounds reduced cell viability in a dose‐dependent manner in EGFR‐high expressing cell lines A431 and HCT116, with estimated half maximal effective concentration (EC_50_) values of 19.7 µM (E151) and 16.7 µM (E152) in A431 cells, and 12.2 µM (both compounds) in HCT116 cells (Figure 7E,F). These results suggest that desloratadine‐based PROTACs can inhibit cancer cell viability. Since E151 had slightly better cell viability effects at higher doses, we primarily utilized this compound for downstream analyses.

PROTACs depend on the ubiquitin‐proteasome system for degradation [36]. We therefore performed rescue experiments to validate the mechanism of action of our compounds. Pre‐treatment with proteasome inhibitor MG132 abolished EGFR degradation by E151, suggesting that proteasomal activity is required (Figure 7G). To implicate E3 ligase activity, we inhibited Cullin‐RING ligase activity with MLN4924, which blocks neddylation and thus activation of the ligase for ubiquitination. Similar to MG132, MLN4924 treatment blocked E151‐mediated degradation, suggesting that neddylation plays a role in the degradation (Figure 7G). Finally, to provide direct evidence for ubiquitination by the E3 ligase, we performed an EGFR immunoprecipitation assay with E151 treatment. MG132 was added to enhance the signal by inhibiting protein degradation, allowing ubiquitinated protein accumulation. In cells expressing HA‐tagged ubiquitin, treatment with E151 increased polyubiquitinated EGFR (Figure 7H). This data suggests that our compounds degrade EGFR through the ubiquitin‐proteasome system.

To further assess the biological activity of our compounds, we tested multiple cancer cell lines with varying levels of EGFR expression. Both E151 and E152 altered the viability of cancer cells upon addition to the culture media (Figure 7I; Figure S9C). E151 showed activity across colorectal cancer cell lines, with estimated EC_50_ values of 30.0 µM in NCI H716 cells, 29.0 µM in SW620 cells, 19.1 µM in COLO205 cells, and 12.2 µM in HCT116 cells. E152 exhibited estimated EC_50_ values of 107 µM in H69 cells, 93.7 µM in HEK293T, 60.6 µM in H3255, and 10.8 µM in A431 cells. In general, the compounds showed selectivity for cell lines expressing higher levels of EGFR like HCT116 and A431, with lower EC_50_ values observed (Figure 7I and Figure S9C). Our data suggests that our compounds have biological effects on cancer cells correlating with EGFR expression.

**FIGURE 8:**
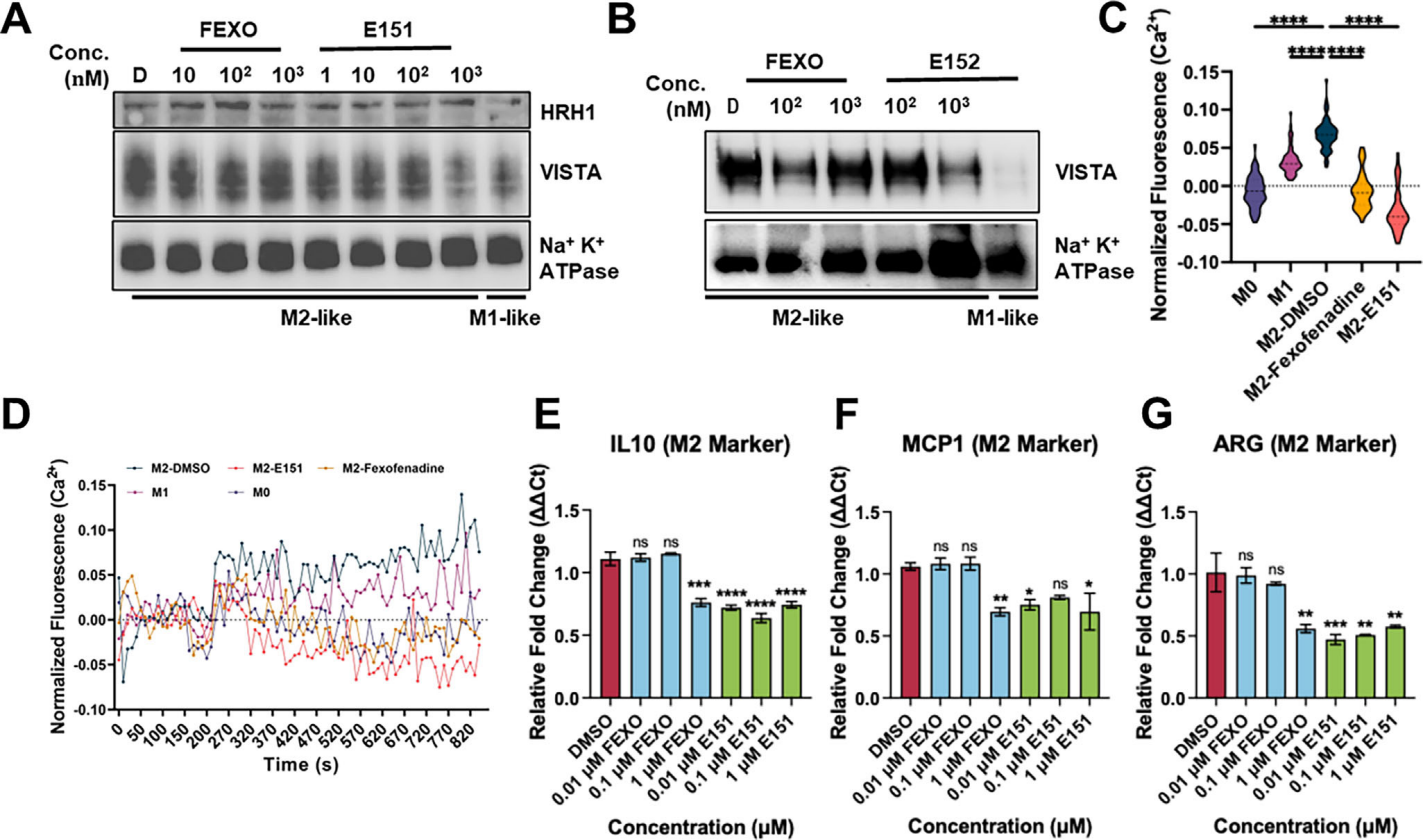
Desloratadine warhead PROTACs inhibit HRH1 to reprogram immune cells. (A, B) Western blot of HRH1, VISTA, and Na^+^/K^+^ ATPase from the membrane protein fraction of M1‐like or M2‐like THP‐1 induced macrophages treated with fexofenadine (FEXO), E151, or E152. (C, D) Mobilization of intracellular calcium (Fluo‐4 assay) of THP‐1 induced M0, M1‐like, and drug treated M2‐like macrophages stimulated with 10 µM histamine stimulation from 180 s, n = 3. The violin plot summarizes the distribution of fluorescence proportional to intracellular calcium intensity. Significance was computed by one‐way ANOVA. (E‐G) qRT‐PCR determined relative mRNA level of M2 macrophage markers (IL‐10, MCP1, ARG) in DMSO‐, fexofenadine‐, or E151‐treated M2‐like macrophages. Significance was computed by one‐way ANOVA.

Although we validated that our PROTACs demonstrated potent EGFR degradation activity, it is unclear if the desloratadine warhead still conferred HRH1 degradation or inhibition activity. Since the immunosuppressive effect of HRH1 occurs on TAMs [25], we examined the effect of our compounds on M2‐like immunosuppressive induced macrophages derived from the human THP‐1 monocytic cell line [37]. We used M1‐like induced macrophages, which are immune responsive, as a control. M1‐like macrophages express less HRH1 than M2‐like macrophages, similar to prior published work (Figure 8A, Figure S10A) [25]. In addition, we validated that the M2 induced macrophages expressed the appropriate functional markers (Figure S10B–F). We then performed membrane protein fractionation after compound treatment to examine membrane‐bound HRH1 levels. Interestingly, E151 did not significantly alter HRH1 protein levels at concentrations up to 1 µM, similar to HRH1 inhibitor fexofenadine [38] (Figure 8A). This data suggests that our PROTACs likely do not degrade HRH1 in induced macrophages.

We next examined the effects of our compounds on the macrophage immune checkpoint pathway. A key mechanism by which M2 macrophages induce immunotherapy resistance is through the increased expression of membrane‐bound checkpoint protein VISTA, which is almost absent in M1 immune responsive macrophages [25]. We therefore examined if our PROTACs had effects on VISTA. Intriguingly, both E151 and E152 treatment on M2‐like induced macrophages could decrease membrane‐bound VISTA levels similar to fexofenadine (Figure 8A,B). This decrease in HRH1 mimics an M1‐like state with low HRH1 expression (Figure 8A,B). It is possible that our PROTACs retain the HRH1 inhibitory ability of desloratadine. HRH1 activation by histamine induces calcium mobilization from the endoplasmic reticulum [39]. We therefore utilized a calcium flux assay to assess HRH1 activity in M0 (uninduced THP1 cells), M1, and M2 induced macrophages. The M0 state has low HRH1 expression and serves as a negative control for the experiment (Figure S10A). As M1‐like macrophages express less HRH1 than M2‐like macrophages, histamine addition showed increased calcium flux in M2 compared to M1 macrophages (Figure 8C,D). Interestingly, both fexofenadine and E151 significantly inhibited calcium flux, suggesting that they inhibited HRH1 activity (Figure 8C,D). These data suggest that our PROTACs inhibit HRH1 to potentially modulate the macrophage immune checkpoint.

To further validate the effects of our compounds on the macrophages, we assessed the levels of various M1 and M2 markers in M2‐like induced macrophages in response to treatment. Both fexofenadine and E151 significantly downregulated M2 markers IL10, MCP1 and ARG (Figure 8E–G). Interestingly, E151 was more effective than fexofenadine at suppressing these markers at similar doses. We observed a similar trend with E152 treatment, which significantly decreased M2 marker levels (Figure S10G–J) and concurrently increased M1 marker levels (Figure S10K,L) in a dose‐dependent manner. This data suggests that our compounds can convert M2‐like induced macrophages to an M1‐like state.

## 3 Discussion

CFAST enables FA to measure cellular proteins‐of‐interest with minimal purification. Since the development of FA as an assay to quantify molecular interactions of biomolecules in the 1950s [40, 41, 42], there have been more than 1000 unique examples of FA assays developed, with the vast majority used for high‐throughput screening [18]. The traditional FA assay typically involves a purified protein target with a fluorescent dye‐labeled probe with known binding affinity to the protein [18]. This limitation precludes the use of FA for quantifying a broad range of proteins and for identifying small molecule binders of undruggable targets. Our CFAST probes are dye‐labeled nanobodies, where the nanobody can be chosen based on one's protein‐of‐interest to be interrogated. In the case where nanobodies against a target of interest is unavailable, one can utilize a tagged version of the protein with a tag‐specific nanobody probe as we have done in our SOX2 and EGFR screens. The modularity of CFAST could therefore broaden the utility of FA for protein quantification and in the case of thermal shift‐CFAST, for probe and drug discovery, against a variety of targets.

In addition to potentially broad utility, CFAST is straightforward, scalable, and cost‐effective. This method requires commonly available lab equipment such as centrifuges, heat blocks, thermocyclers, and FA‐enabled plate readers. All reagents required are commercially available for a reasonable price. We estimate that in a 384‐well screening format, the cost of the assay per sample is about US$0.20. We predict that CFAST will be practical for the majority of research labs, promoting accessible protein quantification as well as PROTAC discovery and development against new targets of interest. We demonstrate this by performing thermal‐shift‐CFAST screens to identify tool compounds against undruggable transcription factor SOX2 and membrane oncogene EGFR.

Transcription factors and membrane proteins are two classes of challenging proteins to drug. Transcription factors comprise the second largest class of oncogene but are ≈85% undruggable [43]. Owing to transcription factors being intrinsically disordered, it is currently difficult to obtain structures or develop in vitro screening assays for effective drug discovery [44]. Similarly, membrane proteins comprise ≈60% of possible drug targets. However, owing to the necessity for membrane localization for structural stability and function, structural determination and screening assay development is likewise challenging [45]. As a proof of concept that thermal shift‐CFAST can address these drug discovery difficulties, we conducted successful screens against undruggable transcription factor oncogene SOX2 and membrane‐bound oncogene EGFR. In terms of SOX2, we posit that our identified tool compound, Wedelolactone, will be useful for developing a targeted SOX2 degrader for probing cancer biology or for drug discovery. With regards to our bifunctional EGFR degrading‐HRH1 inhibiting PROTACs, we suggest that these tool compounds can be useful in understanding the synergy of simultaneously targeting oncogenic signaling and modulating a TME immune checkpoint in EGFR‐dependent cancers. In addition, these compounds could be used to assess the efficacy of a two‐pronged therapy in these cancers. It is interesting to note that our PROTAC likely does not degrade HRH1 but merely inhibits it. Further optimization is required to address if this class of compound can synergistically degrade both EGFR and HRH1. Thermal‐shift‐CFAST therefore provides an opportunity to develop PROTACs against challenging targets.

While CFAST is practical, it has some limitations. Since certain cellular proteins‐of‐interest might be expressed at low levels, it will be useful to enhance the ability of CFAST to robustly detect such proteins without overexpressing them. We observed that the accuracy of CFAST is correlated to higher dye‐labeling efficiency, which results in higher fluorescence intensity values. It could therefore be useful to develop long life‐time fluorophores with higher fluorescence intensities to enable CFAST detection of low abundance proteins. Advances in fluorescence detection instrumentation could also enhance CFAST. In terms of thermal shift‐CFAST, this method is more suited to large scale small molecule library screening on one or a few target proteins, unlike TPP which can screen hundreds to thousands of small molecules on the entire proteome. There is also potential for interference from fluorescent compounds in the assay.

In summary, CFAST provides a versatile FA‐based platform for various protein quantification applications. As an alternative to Western blot‐based detection, CFAST enables rapid protein quantification from cell lysates with minimal purification. Coupled with cellular thermal shift, CFAST also provides a broadly applicable, cost‐effective, and high‐throughput detection method for cellular protein–small molecule interactions. This platform has the potential to facilitate ligand discovery and PROTAC development, particularly for challenging targets that are not amenable to conventional approaches. We propose that CFAST can be a valuable alternative tool for biological research and drug discovery.

## 4 Materials and Methods

### 4.1 Lysis Buffer

Pbs ph 7.2 (gibco #20012027), 5 mm mgcl_2_ (invitrogen #am9530g), 0.01(v/v) triton x‐100 (sigma‐aldrich #t8787), protease inhibitor cocktail (roche #11836170001)

### 4.2 labeling Buffer

PBS pH 7.4 (Gibco #11965092), 5 mM EDTA (Sigma‐Aldrich #E8008), 150 mM NaCl (Sigma‐Aldrich #S6546).

### 4.3 Reagents

Olaparib (Selleck #S1060), Nutlin3 (TOPSCIENCE #T2158), PF‐3644022 (MCE #HY‐107427), A771726 (Sigma‐Aldrich #100128), Pronethalol (MCE #HY‐B1238), DMSO (Aladdin #D103276), Mycophenolic acid (MPA) (Sigma‐Aldrich #475913), Fluzoparib (MCE #HY‐114778), Rucaparib phosphate (MCE #HY‐10617), Veliparib (MCE #HY‐10129), Niraparib (MCE #HY‐10619), Metoclopramide (MCE #HY‐17382), Talazoparib (MCE #HY‐16106), Xipamide (MCE #HY‐W042301), Wedelolactone (MCE # HY‐N0551), Doxapram (MCE #HY‐B0551), Palmitoleic acid (MCE #HY‐W011873), Azamethiphos (MCE #HY‐114899), Desloratadine (Bidepharm #BD123824), (‐)‐Epigallocatechin Gallate (EGCG) (Bidepharm #BD42886), Oxaprozin (Bidepharm #BD19346), Artesunate (Bidepharm #BD22980), Probenecid (TargetMol #T0457), UltraNuclease (Yeason #20156ES60), drug library (Selleckchem #L1300).

### 4.4 Antibodies

ChromoTek PARP1 VHH, recombinant binding protein (Proteintech #xt), ChromoTek GFP VHH, recombinant binding protein (Proteintech #gt), ChromoTek MK2 VHH, recombinant binding protein (Proteintech #mt), ChromoTek Myc VHH, recombinant binding protein (Proteintech #yt), Sox2 Antibody (CST #2748), EGF Receptor (C74B9) Rabbit mAb (CST #2646), PARP1 Polyclonal antibody (Proteintech #13371‐1‐AP), MAPKAP Kinase 2 Antibody (GENXSPAN #GXP314100), GFP (D5.1) Rabbit mAb (CST #2956S), HRH1 rabbit antibody (Proteintech; 28763‐1‐AP), EGFR rabbit antibody (Cell Signaling Technology, 4267), VISTA (Selleck, G23H7), ATPase (Beyotime, AF1864), SOD1(Beyotime, AF8028), HA‐tag (Sigmaaldrich, SAB5600116), GAPDH (Beyotime, AF0006).

### 4.5 Recombinant Proteins

PARP1 Active human (Sigma‐Aldrich #SRP0192), Recombinant Human Active MAPKAPK2 (46‐end) Protein (R&D Systems #3705‐KS), human SOX2 Protein (ReadCrystal #RC‐P1511‐1), human IL‐4 protein (TargetMol, TMPY‐01862), human IL‐13 protein (TargetMol, TMAB‐00049).

### 4.6 Theoretical Fluorescence Polarization Simulation

Theoretical fluorescence polarization curves were generated based on the Perrin equation to illustrate the effect of fluorophore lifetime on molecular weight‐dependent polarization changes. The fluorescence anisotropy (*r*) was calculated according to:

$$r=\frac{r_{0}}{1+\tau /\theta }$$

where *r*_0_ was the fundamental anisotropy (assumed to be 0.4), τ was the fluorescence lifetime of the fluorophore, and *θ* was the rotational correlation time. The rotational correlation time was approximated as proportional to molecular weight for globular proteins:

θ=0.6×MW

where *MW* was the molecular weight in kDa and *θ* was in nanoseconds.

Fluorescence polarization values (mP) were then converted from anisotropy using:

$$P=\frac{2r}{3-r}$$

and expressed in millipolarization units:

mP=P×1000

Simulations were performed using fluorescence lifetimes of 4 ns for FITC and approximately 20 ns for KU DAOTA dye, assuming rigid fluorophore attachment and spherical molecular rotation.

### 4.7 Fluorescence Spectroscopy

The excitation and emission spectra of the DAOTA‐labeled nanobody probe were measured using a microplate reader (Agilent BioTek Synergy Neo2). For emission spectra, samples were excited at 530 nm and emission was recorded over a wavelength range of 400–700 nm. For excitation spectra, emission was monitored at 590 nm while excitation wavelengths were scanned over 400–700 nm. All measurements were performed under assay‐relevant buffer conditions to ensure consistency with CFAST experiments. Samples were measured at room temperature in black 384‐well plates (Greiner #784076) to minimize background fluorescence.

### 4.8 Western Blotting

Membrane protein was extracted from cell samples by Membrane and Cytosol Protein Extraction Kit (Beyotime). Samples were boiled in 5X SDS‐PAGE loading buffer (Beyotime) at 95°C for 10 mins. The sample can be frozen and stored at ‐20°C for later use. Proteins were separated by Express Plus PAGE Gels (GenScript) and transferred to a Mini‐size PVDF membrane (TransBlot Turbo). Blocking and antibody incubation were performed with Beyotime Quick Block, according to the manufacturer's instruction. Antibody detection was carried out by Clarity Western ECL Substrate (BIO‐RAD).

### 4.9 Surface Plasmon Resonance Experiments

Surface plasmon resonance experiments were performed on the Biacore 8K instrument (Cytiva). Recombinant SOX2 (Readcrystal) was immobilized at 25°C on CM5 Chips (Cytiva) in sodium acetate (pH 4.5 Cytiva). The surface was activated using 400 mM 1‐ethyl‐3‐(3‐dimethylaminopropyl)‐carbodiimide and 100 mM N‐hydroxysuccinimide (Cytiva) (contact time 120 s, flow rate 30 mL/min). The surface was subsequently deactivated by injecting 1 M ethanolamine for 100 s and conditioned by injecting 50 mM NaOH. Dilution of the SOX2 target protein and coupling was performed using a running buffer without DMSO. The SOX2 target proteins were prepared at 50 µg/mL and coupled to the chip to a density between 7149 response units. The compounds were diluted in running buffer and injected over the immobilized target proteins (concentration range, 0.125‐12.5 µM). Sensorgrams from reference surfaces and blank injections were subtracted from the raw data before data analysis using Biacore Insight software. Affinity and binding kinetic parameters were determined by using a 1/1 interaction model, with a term for mass transport included.

### 4.10 Cell Culture

Human embryonic kidney 293T cells (HEK293T cells, ATCC CRL‐3216), HEK293T Tg:MK2 and HEK293T Tg:GFP were cultured in Dulbecco's Modified Eagle Medium (Gibco); Hela (ATCC CRM‐CCL‐2) and Hela KO PARP (Abclonal) were cultured in Dµlbecco's Modified Eagle Medium; H69 (ATCC HTB‐119) were cultured in Roswell Park Memorial Institute (RPMI) 1640 All culture media contain 0.1 g/L L‐glutamine and 10% fetal bovine serum (FBS, AusGeneX, Australia), 100 units/mL penicillin and streptomycin (Gibco).

### 4.11 Heterologous Protein Expression

To generate stable HEK293T *Tg:MK2* and HEK293T *Tg:SOX2/EGFR*‐2*Myc‐tag cell line, HEK293T cells were cultured in DMEM medium supplemented with 10% FBS and 1X penicillin/streptomycin. 70% confluent HEK293T cells were transfected in Opti‐MEM (#31985‐088, Gibco) using 3.0 µg of the pCDH‐CMV‐Homo‐MK2‐EF1a‐puro (IGEbio) or pCDH‐CMV‐Homo‐SOX2‐2*Myc‐tag‐EF1a‐puro (IGEbio), 1.5 µg pMD2.G (#12259, Addgene), 1.5 µg psPAX2 (#12260, Addgene), and 13 µL of Lipo2000 (#11668019, Invitrogen) as per protocol. Cells were incubated for 4 hrs and media was replaced. Virus was collected 48 hrs post transfection and filtered through 0.45 µm filter (Merck Millipore), fresh media was added for second viral harvest. Lenti‐concentrate virus precipitation (EMB810A‐1, Excel Bio) was added and kept overnight at 4°C. The next day, the virus pellet was collected by centrifugation at 3000 rpm, 30 min, 4°C. After centrifugation, the supernatant was removed, and viral pellet was resuspended in ice cold supplemented media. Concentrated virus was added to the desired cell line with polybrene 8 ug/ml. Puromycin selection was started at 48 hrs post‐transduction. After 7 days, cells were collected and allowed to recover for three additional days before further analysis.

### 4.12 CFAST Probe Synthesis

A. N‐Methyl‐N‐(4‐malimide‐butyl)‐DAOTA‐maleimide dye (KU‐dyes, KU560‐R‐6)

Wet the ultrafiltration tube with cold labeling buffer at 5000 rcf for 1.5 hrs at 4°C. Add 125 µL nanobody and fill with cold labeling buffer. After mixing, centrifuge with the above conditions. Incubate nanobody and DAOTA‐5‐maleimide reaction for 2 hrs at room temperature. Remove unreacted DAOTA by processing with dye removal columns (Thermo Scientific #22858). Store labeled nanobody at 4°C for up to one month away from light.

B. N‐Methyl‐N‐(4‐malimide‐butyl)‐DAOTA‐NHS dye (KU‐dyes, KU560‐R‐4)

Wet the ultrafiltration tube with cold PBS (pH7.4) at 5000 rcf for 1.5 hrs at 4°C. Add 125 µL nanobody and fill with cold PBS. After mixing, centrifuge with the above conditions. Incubate nanobody and DAOTA‐NHS reaction for overnight at 4°C. Remove excess DAOTA by dye removal columns (Cytiva #28918007). Store labeled nanobody at 4°C for up to one month away from light.

### 4.13 CFAST Protocols

#### 4.13.1 CFAST Workflow for Cellular Endogenous Protein Quantification (1.5 ml Tube)

1.5 × 10^7^ cells were washed with PBS twice. The remaining PBS was then removed after 400 g centrifugation for 5 min. Cell pellets were re‐suspended with 300 µL of lysis buffer. Cell suspensions were freeze‐thawed three times with liquid nitrogen to lyse the cells. Cell lysate was incubated with UltraNuclease (2 µL per 1 × 10^7^cells) for 1.5 hrs at 37°C. Next, the lysate was centrifuged at 20,000 g for 20 mins at 4°C. The supernatants were transferred to a 384 well plate (Greiner #784076), 10 µL/well. Probe was diluted with lysis buffer (1:500–1:1000) and 5 µL diluted probe was added per well. Mix by gently pipetting and incubate for 10 mins. Fluorescence anisotropy (FA) was detected with a plate reader (Agilent BioTek Synergy Neo2 #N2MABT‐SN) 8 times per well and FA was then measured with (EX 530/25, EM 590/35) filters for each sample. The average mP value was plotted for each replicate well.

#### 4.13.2 Thermal Shift‐CFAST Workflow for Detecting Cellular Protein‐Small Molecule Interactions (1.5 ml Tube)

Equal numbers of cells (1.5 × 10^7^) were seeded in 6‐well cell culture plates (Biofil #TCP 011006) with the appropriate amount of cell culture medium and incubated with drug for 2 hrs in an incubator. After the cells were harvested, they were washed twice with PBS to remove excess drug and culture medium. Remaining PBS was then removed after 400 rcf centrifugation for 5 min. Cell pellets were re‐suspended with 300 µL of lysate buffer. Heat samples at their target‐dependent temperatures in a thermocycler (Bio‐Rad, CFX96 Deep Well Dx). For PARP1, heat samples at 50°C for 3 mins and then 4°C for 3 mins. For MK2, heat samples at 54°C for 3 mins and then 4°C for 3 mins. For SOX2, heat samples at 58°C for 5 mins and then 4°C for 3 mins. Next, cell suspensions were freeze‐thawed three times with liquid nitrogen to lyse the cells. Cell lysate was incubated with UltraNuclease (2 µL per 1 × 10^7^ cells) for 1.5 hrs at 37°C. Next, the lysate was centrifuged at 20000 g for 20 mins at 4°C to remove insoluble material. The supernatants were transferred to a 384 well plate (Greiner #784076), 10 µL/well. Probe was diluted with lysis buffer (1:500‐1:1000) and 5 µL diluted probe was added per well. Mix by gently pipetting and incubate for 10 min. Detect FA with plate reader (Agilent BioTek Synergy Neo2 #N2MABT‐SN) 8 times per well and FA was then measured with (EX 530/25, EM 590/35) filters for each sample. The average mP value was plotted for each replicate well.

#### 4.13.3 Thermal Shift‐CFAST Workflow for High‐Throughput Screening (384‐Well Plate)

Equal numbers of cells (1 × 10^6^) were seeded in 384‐well PCR plates (Biorad #HSP3801) with 25 µL cell culture medium and incubated with drug for 2 hrs in an incubator. After the cells were harvested, they were washed twice with PBS to remove excess drug and culture medium. Remaining PBS was then removed after 400 rcf centrifugation for 5 mins. Cell pellets were re‐suspended with 30 µL of lysis buffer. After heating at the target‐dependent temperature in a thermocycler for 6 min, the cell suspensions were freeze‐thawed three times with liquid nitrogen to lyse the cells. Cell lysate was incubated with UltraNuclease (2 µL per 1 × 10^7^cells) for 1.5 hrs at 37°C. Next, the lysate was centrifuged at 4000 rpm for 60 mins at 4°C to remove insoluble material. The supernatants were transferred to a 384 well plate (Greiner #784076), 10 µL for experimental probe and another 10 µL for GFP probe. Probe was diluted with lysis buffer (1:500–1:1000) and 5 µL diluted probe was added per well. Mix by gently pipetting and incubate for 10 mins. FA was detected with a plate reader (Agilent BioTek Synergy Neo2 #N2MABT‐SN) 8 times per well and FA was then measured with (EX 530/25, EM 590/35) filters for each sample. The average mP value was plotted for each replicate well.

### 4.14 Chemical Synthesis

*tert*‐butyl (2‐(2,6‐dioxopiperidin‐3‐yl)‐1,3‐dioxo‐2,3‐dihydro‐1*H*‐inden‐5‐yl)glycinate (1)

*tert*‐butyl 3‐((2‐(2,6‐dioxopiperidin‐3‐yl)‐1,3‐dioxo‐2,3‐dihydro‐1*H*‐inden‐5‐yl)amino)propanoate (2)

(2‐(2,6‐dioxopiperidin‐3‐yl)‐1,3‐dioxo‐2,3‐dihydro‐1*H*‐inden‐5‐yl)glycine (1a)

3‐((2‐(2,6‐dioxopiperidin‐3‐yl)‐1,3‐dioxo‐2,3‐dihydro‐1*H*‐inden‐5‐yl)amino)propanoic acid (2a)

4‐Fluorothalidomide (124 mg, 0.45 mmol, 1.0 equiv.), amine (0.50 mmol, 1.1 equiv.) and DIPEA (230 µL, 1.35 mmol, 3.0 equiv.) were dissolved in DMSO (1.0 mL, 0.2 M) and warmed to 130°C for 12 hrs. The residue was purified by flash column chromatography over silica gel, eluted with EtOAc:hexanes (20 –100%) to afford a yellow viscous oil (1‐4, 80–90%), then dissolved in 4 ml TFA for 4 hrs to achieve 1a‐4a (90%).

3‐(5‐((2‐(4‐(8‐chloro‐5,6‐dihydro‐11*H*‐benzo[5,6]cyclohepta[1,2‐*b*]pyridin‐11‐ylidene)piperidin‐1‐yl)‐2‐oxoethyl)amino)‐1,3‐dioxo‐2,3‐dihydro‐1*H*‐inden‐2‐yl)piperidine‐2,6‐dione (E151)

3‐(5‐((3‐(4‐(8‐chloro‐5,6‐dihydro‐11*H*‐benzo[5,6]cyclohepta[1,2‐*b*]pyridin‐11‐ylidene)piperidin‐1‐yl)‐3‐oxopropyl)amino)‐1,3‐dioxo‐2,3‐dihydro‐1*H*‐inden‐2‐yl)piperidine‐2,6‐dione (E152)

### 4.15 Fluorescence Polarization

5‐FAM (Bidepharm, BD243928) labeled desloratadine (DL‐1, 10 nM) was mixed with increasing concentrations of either recombinant EGFR protein (Sigma, SRP0239) or CA II (MCE, HY‐P72860) (0.002–1.5 µM) in a 384‐well microplate (Greiner, 784076) and incubated for 30 min at room temperature. EGFR‐DL‐1 interactions were measured in 50 mM Tris pH 7.5, 100 mM NaCl, 0.1% TCEP and 1% DMSO via fluorescence polarization using the BIOTEK NEO2 microplate reader. Data were plotted and analyzed using GraphPad Prism 10.

### 4.16 Cell Viability

Cells were seeded in 96‐well plates and treated with E151 or E152 in a variety of doses (0.5‐100 µM) after overnight incubation. Cell viability was measured by CellTiter‐Glo 2.0 assay (Promega). Luminescence readout (Agilent BioTek Synergy Neo2 #N2MABT‐SN) from drug treated wells were normalized against control wells and expressed as percentage cell viability.

### 4.17 Macrophage Differentiation

THP‐1 monocytic cells were differentiated into macrophages by 24 hr incubation with 150 nM phorbol 12‐myristate 13‐acetate (PMA, TargetMol, TQ0198) followed by 24 hr incubation in RPMI medium. M1‐like macrophages were obtained by incubation with 20 ng/ml of IFN‐γ (TargetMol, TMPY‐01714) and 10 pg/ml of LPS (TargetMol, T11855). M2‐like macrophages were obtained by incubation with 20 ng/ml of interleukin 4 (IL‐4) and 20 ng/ml of interleukin 13 (IL‐13).

### 4.18 qRT‐PCR

Total RNA was extracted using the HiPure Total RNA Kit (Magen, R4111‐03). 2 µg total RNA was reverse transcribed using the RevertAid First Strand cDNA Synthesis Kit (Thermo Fisher, K1622). Amplification reaction assays contained 2x SYBR Green qPCR Master Mix (Selleck, B21202) and primers (IGE, 300 nM). ACTB was used as the reference gene for normalization and mRNA abundance was quantified using the threshold cycle method.

**Table:** 

Primers	Forward sequence 5’‐3’	Reverse sequence 5’‐3’
HRH1	GCTGGGCTACATCAACTCCAC	CCCTTAGGAGCGAATATGCAGAA
TNFalpha	CTCTTCTGCCTGCTGCA CTTTG	ATGGGCTACAGG CTTGTCACTC
CXCL10	GAAAGCAGTTAGCAAG GAAAGGTC	ATGTAGGGAAGT GATGGGAGAGG
CD206	CCATGGACAATGCGCG AGCG	CACCTGTGGCCC AAGACACGT
CD163	TTTGTCAACTTGAGTCC CTTCAC	TCCCGCTACACT TGTTTTCAC
GAPDH	GCACCGTCAAGGCTGAGAAC	TGGTGAAGACGCCAGTGGA
TGF beta 1 (TGFB)	TACCTGAACCCGTGTTGCTCTC	GTTGCTGAGGTATCGCCAGGAA
Arginase 1 (ARG1)	TCATCTGGGTGGATGCTCACAC	GAGAATCCTGGCACATCGGGAA
MCP1 (CCL2)	AGAATCACCAGCAGCAAGTGTCC	TCCTGAACCCACTTCTGCTTGG
IL10	TCTCCGAGATGCCTTCAGCAGA	TCAGACAAGGCTTGGCAACCCA

### 4.19 Intracellular Calcium Measurement

Intracellular Ca2+ was determined using the Fluo‐4 Calcium Assay Kit (Beyotime) according to the manufacturer's instructions. Fluorescence was measured using a plate reader (Agilent BioTek Synergy Neo2 #N2MABT‐SN).

### 4.20 Statistical Analysis

Statistical analyses were performed using GraphPad Prism 10 (GraphPad Software). Data were presented as mean ± SD unless otherwise indicated. Sample sizes (n) represent biologically independent experiments unless otherwise stated. Statistical tests used for each experiment were specified in the corresponding figure legends. P < 0.05 was considered statistically significant. Statistical significance was indicated as follows: ns, not significant; *P < 0.05; **P < 0.01; ***P < 0.001; ****P < 0.0001.

## Author Contributions

Conceptualization: **J.L.T**., **Q.T**.; Methodology: **Q.T**., **Y.W**.; Investigation: **Q.T**., **Y.W**., **R.M**., **N.W**., **T.Z.Z**., **H.K.H**., **Z.I.B**., **I.I**.; Supervision: **X.L**., **J.W.P.Y**., **Y.H.W**., **Q.W**., **J.L.T**.; Writing – original draft: **Q.T**., **Y.W**., **R.M**., **J.L.T**.; Writing – review & editing: **Q.T**., **J.L.T**.

## Funding

This work was supported by the Shenzhen Bay Laboratory Open Fund, SZBL2021080601003; Shenzhen Bay Laboratory Proof of Concept Grant S231801006 (J.L.T.).

## Conflicts of Interest

J.L.T., Q.T., and Y.W. are co‐inventors on a patent application filed by Shenzhen Bay Laboratory relating to work in this manuscript. The remaining authors disclose no conflicts.

## Supporting information




**Supporting File**: advs76141‐sup‐0001‐SuppMat.docx.

## Data Availability

The data that supports the findings of this study are available in the supplementary material of this article.
