# Supplementary material for: Versatile Detection of Cellular Protein via Fluorescence Anisotropy
Source: Adv Sci (Weinh). 2026 Jul 13;13(51):e76141. doi: 10.1002/advs.76141 (PMC13360121; doi:10.1002/advs.76141)
Supplement: Supplementary file 1 [file ADVS-13-e76141-s001.docx]

Supplemental Figures:

**Fig. S1. Nanobody probes provide improved FA dynamic range compared to conventional antibodies for protein detection. (A)** Simulated relationship between FA and molecular weight, illustrating the expected change in anisotropy upon binding of MK2 protein to a nanobody (VHH, ~14 kDa) or an antibody (~150 kDa) probe. The smaller size of the nanobody probe results in a larger anisotropy change (ΔmP) upon target binding compared to an antibody-based probe. **(B)** Fluorescence excitation and emission spectra of free DAOTA dye measured under assay conditions, with excitation and emission maxima at ~550 nm and ~590 nm respectively. **(C)** FA measurements in the presence or absence of recombinant MK2 protein combined with either DAOTA-labeled nanobody (VHH) or antibody probes specific to MK2. Data are presented as mean ± SD, n = 4. Statistical significance was determined using a two-tailed unpaired Student’s t-test.

**Fig. S2. CFAST probes bind to their protein targets.** **(A)** FA measurements of PARP1 and EGFP probes with varying concentrations of recombinant PARP1 protein. **(B)** FA measurements of PARP1, MK2, and EGFP probes with varying concentrations of recombinant MK2 protein. ΔmP values were obtained by normalizing to mP values of the corresponding 0.001 nM protein sample. Data are presented as mean ± SD, n = 2. Statistical significance was determined using a two-tailed unpaired Student’s t-test.

**Fig. S3. Nuclease incubation reduces background.** FA measurements of MK2 probe in HEK293T and HEK293T expressing MK2 cell lysate, with and without heat and nuclease treatment. ΔmP values were obtained by normalizing to FA measurements of EGFP probe added to aliquots of the experimental samples. Data are presented as mean ± SD, n = 5. Statistical significance was determined using a two-tailed unpaired Student’s t-test.

**Fig. S4. CFAST workflow for cellular protein quantification (1.5 ml tube).** Created with BioRender.

**Fig. S5. The long lifetime DAOTA fluorophore improves FA dynamic range compared to short lifetime FITC. (A)** Simulated relationship between FA and molecular weight for probes labeled with fluorophores of different fluorescence lifetimes. DAOTA (long lifetime) exhibits a larger dynamic range of anisotropy change (ΔmP) compared to FITC (short lifetime), particularly for proteins in the molecular weight range relevant to many cellular targets. **(B)** FA measurements of FITC-labeled PARP1 and GFP nanobody probes in Hela and Hela PARP1 knockout cell lysates. Data are presented as mean ± SD, n = 4. Statistical significance was determined using a two-tailed unpaired Student’s t-test

**Fig. S6. CFAST workflow for detecting cellular protein-small molecule interactions (1.5 ml tube).** Created with BioRender.

**Fig. S7. CFAST workflow for high-throughput screening (384-well plate).** Created with BioRender.

**Fig. S8. Validation of desloratadine as an EGFR binder.** **(A)** Fluorescence polarization assay of fluorescent dye-labeled desloratadine with purified EGFR or CAII protein, n = 6. **(B)** Temperature gradient CETSA Western blot of EGFR and SOD1 in DMSO- and desloratadine-treated A431 cells. **(C)** Quantified EGFR protein levels in **(B)**. **(D)** Dose-response CETSA Western blot of EGFR and SOD1 in DMSO- and desloratidine-treated A431 cells, heated at 64 ℃.

**Fig. S9. Assessment of desloratadine and EGFR PROTAC activity.** Dose-response western blot of EGFR and Actin in DMSO- and desloratadine-treated **(A)** A431 cells and **(B)** HCT116 cells. **(C)** Cell viability of E152 treatment in various cancer cell lines with variable EGFR expression, measured by CellTiter-Glo assay. Data are presented as mean ± SD, n = 3.

**Fig. S10. PROTACs E151 and E152 alter macrophage subtype gene expression signatures.**  **(A-F)** qRT-PCR determined relative mRNA level of HRH1 and M2 macrophage markers (CD206, IL-10, ARG, MCP1, and TGFB) in human monocyte THP-1 induced M1-like or M2-like macrophages. Significance was computed by one-way ANOVA. **(G-L)** qRT-PCR determined relative mRNA level of M2 macrophage markers (IL-10, ARG, TGFB, and CD163) and M1 macrophage markers (TNFalpha and CXCL10) in DMSO-, fexofenadine-, or E152-treated induced M2-like macrophages. Significance was computed by one-way ANOVA.
